# Epidemiology of lactic acidosis in type 2 diabetes patients with metformin in Japan

**DOI:** 10.1002/pds.4030

**Published:** 2016-05-25

**Authors:** Chia‐Hsien Chang, Motonobu Sakaguchi, Paul Dolin

**Affiliations:** ^1^ Takeda Development Center JapanTakeda Pharmaceutical Company LimitedOsakaJapan; ^2^ Takeda Development Centre Europe LtdLondonUnited Kingdom

**Keywords:** metformin, lactic acidosis, Japan, MDV database, pharmacoepidemiology

## Abstract

**Purpose:**

To estimate the incidence of lactic acidosis (LA) and role of metformin in Japanese patients with type 2 diabetes mellitus (T2DM) treated with anti‐diabetes drugs.

**Methods:**

This retrospective propensity score matched cohort study was conducted using the Japanese Medical Data Vision claims database. T2DM patients aged 18 or above who received diabetes drugs during January 2010 through August 2014 were identified. Cases of LA were identified based on reimbursement codes and confirmed by lactic acid test and subsequent treatment by hemodialysis or intravenous sodium bicarbonate. Poisson regression and Cox proportional hazard models were used to estimate the incidence and assess if metformin use was associated with increased risk of LA.

**Results:**

Thirty cases of LA were identified among 283 491 treated T2DM patients with 504 169 patient‐years of follow‐up. Crude incidence of LA was 5.95 per 100 000 patient‐years. T2DM patients with chronic kidney disease (CKD) were seven‐fold more likely to develop LA than those without CKD (adjusted hazard ratio (aHR), 7.33, 95%CI, 3.17–16.96). Use of metformin was not associated with risk of LA in the study population (aHR, 0.92, 95%CI, 0.33–2.55), and in the propensity score matched cohort (aHR, 0.90, 95%CI, 0.26–3.11). Similar findings were observed among diabetes patients with chronic liver disease (CLD) and CKD. The age‐sex adjusted incidence rates in metformin users and non‐users were 5.80 and 5.78 per 100 000 person‐years, respectively (Incidence rate ratio, 1.00, *p* = 0.99).

**Conclusions:**

This study found that use of metformin was not associated with increased risk of LA in diabetic patients including those with CKD or CLD. © 2016 The Authors. Pharmacoepidemiology and Drug Safety published by John Wiley & Sons Ltd.

## Introduction

Lactic acidosis (LA) is a life‐threatening condition characterized by low arterial pH (<7.35) and elevated arterial lactate levels (>5.0 mEq/l). This critical illness usually coexists with serious medical conditions such as sepsis, hypoxia and cardiac failure.^1^ The liver plays a major role in gluconeogenesis, and hepatic failure can dramatically hamper lactate removal. An early biguanide, phenformin, was withdrawn from the market because of a reported LA rate of 40 to 64 cases per 100 000 patient‐years.^2^, ^3^ Use of metformin is contraindicated in diabetic patients with renal failure or renal dysfunction (creatinine clearance < 60 ml/min) because of the concern on the accumulation of metformin and risk of LA.^4^ Reported cases of LA acidosis in patients on metformin have occurred primarily in diabetic patients with significant renal failure.^5^, ^6^ However, metformin has a different molecular structure and pharmacokinetics from phenformin, and can enhance glucose oxidation without significantly affecting fasting lactate production in peripheral tissues.^7^

A Cochrane review of 347 comparative trials and cohort studies showed no cases of fatal or nonfatal LA in 70 490 patients‐years of metformin use or in 55 451 patients‐years in the non‐metformin group.^5^ Studies of lactate concentrations in patients with T2DM showed a low correlation between serum metformin levels and lactate concentration.^8^ There is limited evidence that metformin use in the absence of renal disease is associated with an increased risk of LA.^5^, ^8^ The available evidence suggests that metformin use only increases risk of LA in the presence of other concomitant contributing factors such as renal failure or renal insufficiency.^7^ Little data is currently available to determine the relationship between metformin use, renal disease and LA.

This study was conducted to estimate the incidence of LA among Japanese T2DM patients treated with anti‐diabetic medications and determine the role of metformin. We also look at the role of metformin in LA among patients who are at risk of metformin accumulation such as those with chronic liver disease (CLD), CKD and the elderly.

## Methods

This retrospective propensity score matched cohort study was conducted using the Japanese Medical Data Vision (MDV) database. MDV is a commercial, electronic, record‐based healthcare database that contains patient level information on demographic characteristics, diagnoses and prescription information (dose, quantity and number of days of supply) for approximately 8 140 000 patients at 153 medical facilities across Japan up to 2014. The age and gender distribution of patients in the database is similar to that of the national demographics for seeking healthcare.^9^ Patients' identities were encrypted for protection of privacy.

The study cohort included all patients aged 18 or above with at least one claims for an anti‐diabetes prescription (ATC code: A10A, A10B), and excluded any patients with a diagnosis of type 1 diabetes anytime (ICD‐10 code, E10) during the study period 1 January 2010 to 31 August 2014. The first prescription of metformin or other diabetes medications during the study period served as the index date. We excluded patients with a diagnosis of LA during 180 days before index date, and patients with less than 30 days of continuous diabetes treatment after index date. Within study cohort, patients were eligible to be new user if they had at least 180 days of enrollment in the MDV database prior to first diabetes medications.

To account for imbalance in baseline characteristic between these two groups, a propensity score was calculated and constructed 20 strata of 5% each for the distribution scores, based on the baseline variables, and each metformin user was matched to the non‐metformin user with nearest propensity score, without replacement.^10^

### Exposure definitions and measures

We calculated the cumulative duration of metformin (ATC code: ‘A10BA02’, ‘A10BD05’) use by summing each day's supply for all prescriptions starting at the index date, with allowance for 90‐day gaps in therapy. If a single large dose was recorded in the patient record for patients monitored regularly, we estimated duration of the supply by dividing the large single dose by previous daily dose for that patient (suggested dosage for metformin in Japan: 500–2250 mg per day).^11^ Patients using both metformin and another diabetes drugs were classified as metformin user. Patients were followed‐up throughout study period, and exposure to metformin was treated as a time‐varying variable.

### Outcome definitions and measures

A two‐stage strategy was used to identify cases of LA, as there was no specific ICD‐10 code for LA. The database contained the original diagnosis as written in the Japanese language. We first identified all records of LA using Japanese language computer‐assisted text searching. All potential LA cases were then required to have a lactic acid test within 30 days before the diagnosis, and to have received hemodialysis or intravenous bicarbonate infusion within 30 days after diagnosis. Although treatment of acidosis with sodium bicarbonate is a matter of controversy,^12^ this treatment does indicate need for metabolic correction. We did not include hemodialysis cases if they had chronic renal failure to avoid misclassification. Patients were followed‐up until the first record of the LA, the patients' last record in the database or the end of study period, which ever occurred first.

### Statistical methods

Descriptive statistics, including means (standard deviation, SD) for continuous variables and number (percentage, %) for category variables, were used to describe baseline characteristics. The incidence rate for LA calculated per 100 000 person‐years with 95% confidence interval (CI) and adjusted incidence rate ratio were estimated by Poisson regression. Cox proportional hazard regression analysis was used to account for time‐varying exposure and generate LA hazard estimates for metformin use.

History of medical conditions related to diabetes treatment including hypertension, ischemic heart disease, heart failure, complications of diabetes, dyslipidemia, cerebrovascular disease, biliary disease, gastric ulcer, obesity, malignancy, metastatic cancer, CLD and chronic kidney disease was considered present at index date if patients had a diagnostic record within 365 days before the index date. The diagnoses of CLD or CKD were categorized as non‐advanced or advanced, respectively. The advanced stage defined as their disease progress to liver cirrhosis, liver failure, malignant hepatocellular carcinoma, renal failure or uremia. Because the diagnoses for specific CKD stage were very rarely used, it was not possible to classify CKD stage.

Metformin exposure, history of liver disease and history of renal disease and the stratum estimated by propensity score were included in all regression models. Covariates were considered one at a time for confounder if their inclusion resulted in at least a 10% shift in the risk estimate for the base model. Confounding variables which were not missing for most of the patients and also met the 5% significance level were retained in the final model.^13^ Interaction term was also assessed, use the same criteria as for confounding variables. Along with other initial adjustment variables, we only included the stratum estimated by propensity score in the model for propensity score‐matching analysis.

We estimated the rate ratio associated with metformin use from the Poisson regression. The use of metformin was updated in each 90‐day period throughout follow‐up. A sensitivity analysis included separate analyses for new user and sub‐cohorts stratified by history of CLD and CKD. All analyses were performed using SAS 9.3 (SAS Institute, Cary, NC).

## Results

**Figure 1:**
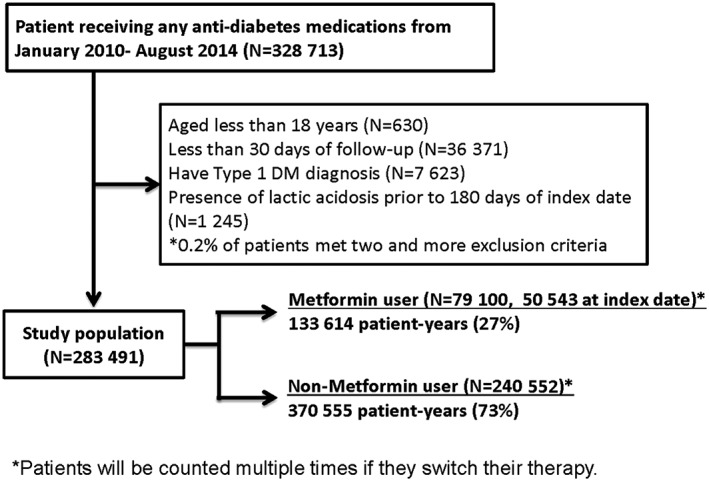
^*^Patients were counted multiple times if they switched their therapy

The study population included 283 491 patients, with a total follow‐up of 504 169 patient‐years (Figure 1). Near 60% of patients enrolled after 2012. A total of 50 543 (18%) patients were treated with metformin, and 232 948 (82%) received other diabetes drugs at index date. A total of 59 869 (21%) patients in the cohort had a diagnosis of CLD, and 47 052 (17%) patients had CKD. The majority of patients were aged 65 years or older (65%) on their index date. Most were male (61%), and the mean age of females was 3 years older than that of males. The majority of patients had sought their healthcare from the large hospitals that provide acute care, and 40% were treated with insulin. Moreover, 4% of patients had a high Charlson comorbidity index (CCI) score of 6 or more. The most frequent comorbidities were hypertension (32%) and complications of diabetes mellitus (16%). The patients who did not use metformin on the index date were more likely to be older, use insulin and higher score of CCI. After propensity score‐matching, the imbalance of comorbidities and use of insulin disappeared (Table 1).

**Table 1:** Baseline characteristics of treated type 2 diabetes mellitus patients—classified by the first use of diabetes mellitus drug on the index date

	Main cohort		Propensity score‐matched cohort	
	Metformin users (*N* = 50 543)	Non‐metformin DM drug user (*N* = 232 948)	Metformin users (*N* = 50 541)	Non‐metformin DM drug user (*N* = 50 541)
Enrollment year				
2010–2011	24 255 (48%)	97 283 (42%)	24 254 (48%)	23 469 (46%)
2012–2013	21 729 (43%)	106 337 (46%)	21 728 (43%)	21 740 (43%)
2014	4559 (9%)	29 328 (12%)	4559 (9%)	5332 (11%)
Gender				
Male	30 428 (60%)	143 865 (62%)	30 428 (60%)	30 441 (60%)
Female	20 115 (40%)	89 083 (38%)	20 113 (40%)	20 100 (40%)
Mean follow‐up period, (SD) year/month	2.1 (1.5)/21 (18)	1.7 (1.4)/21 (17)	2.1 (1.5)/26 (18)	2.0 (1.4)/24 (17)
Age on index date				
Mean age (SD)	63 (12)	69 (12)	63 (12)	65 (12)
Mean age, male (SD)	62 (12)	69 (12)	62 (12)	64 (11)
Mean age, female (SD)	65 (12)	71 (13)	65 (12)	67 (12)
Age groups, stratified by 10 years				
18–34 years	1051 (2%)	2438 (1%)	1050 (2%)	701 (1%)
35–44 years	3270 (6%)	7327 (3%)	3269 (6%)	2200 (4%)
45–54 years	7020 (14%)	16 922 (7%)	7020 (14%)	5125 (10%)
55–64 years	14 640 (29%)	47 932 (21%)	14 640 (29%)	13 858 (27%)
65–74 years	15 629 (31%)	73 926 (32%)	15 629 (31%)	19 746 (39%)
75– years	8933 (18%)	84 403 (36%)	8933 (18%)	8911 (18%)
Prior use of metformin before cohort entry	4437 (9%)	509 (0%)	4436 (9%)	131 (0%)
Diabetes treatment on index date				
Metformin	50 543 (100%)	0	50 541 (100%)	0
Sulfonylurea	21 886 (43%)	67 330 (29%)	21 886 (43%)	19 342 (38%)
DPP‐4 inhibitor	14 481 (29%)	73 215 (31%)	14 481 (29%)	19 437 (38%)
Alpha‐glucosidase inhibitor	11 909 (24%)	50 008 (21%)	11 909 (24%)	13 763 (27%)
TZD	8485 (17%)	26 121 (11%)	8485 (17%)	8323 (17%)
GLP‐1/SGLT2 inhibitors	597 (1%)	2229 (1%)	597 (1%)	785 (2%)
Insulin	8395 (17%)	114 677 (49%)	8395 (17%)	8479 (17%)
Comorbidities				
Hypertension	10 800 (21%)	80 684 (35%)	10 800 (21%)	10 973 (21%)
Ischemic heart disease	3438 (7%)	33 798 (15%)	3438 (7%)	3414 (7%)
Heart failure	2383 (5%)	29 304 (13%)	2383 (5%)	2373 (5%)
Complication of diabetes	7579 (15%)	38 946 (17%)	7577 (15%)	7513 (15%)
Dyslipidemia	10 585 (21%)	50 140 (22%)	10 584 (21%)	10 571 (21%)
Cerebrovascular disease	3395 (7%)	29 993 (13%)	3395 (7%)	3373 (7%)
Biliary disease	1160 (2%)	12 660 (5%)	1160 (2%)	1109 (2%)
Gastric ulcer	3055 (6%)	29 472 (13%)	3055 (6%)	3046 (6%)
Obesity	446 (1%)	1078 (0%)	444 (1%)	391 (1%)
Malignancy	1967 (4%)	35 598 (15%)	1967 (4%)	1881 (4%)
Metastatic cancer	482 (1%)	7759 (3%)	482 (1%)	466 (1%)
Chronic liver disease	11 074 (22%)	48 795 (21%)	11 073 (22%)	11 032 (22%)
Liver cirrhosis	517 (1%)	7696 (3%)	517 (1%)	499 (1%)
Chronic kidney disease	7007 (14%)	40 045 (17%)	7005 (14%)	7017 (14%)
Renal failure	639 (1%)	13 224 (6%)	639 (1%)	638 (1%)
Charlson comorbidity index, (SD)	0.8 (1.3)	1.7 (1.9)	0.8 (1.3)	0.9 (1.3)
Score 0	32 398 (64%)	86 551 (37%)	32 398 (64%)	29 942 (59%)
Score 1	6634 (13%)	39 593 (17%)	6634 (13%)	8061 (15%)
Score 2	6966 (14%)	41 922 (18%)	6965 (14%)	7173 (14%)
Score 3	2554 (5%)	28 079 (12%)	2554 (5%)	2851 (6%)
Score 4	1007 (2%)	16 142 (7%)	1006 (2%)	1330 (3%)
Score 5	482 (1%)	9152 (4%)	482 (1%)	622 (1%)
Score ≥ 6	502 (1%)	11 509 (5%)	502 (1%)	562 (1%)

We identified 30 patients meeting the case definition of LA. The majority were at least 65 years old when the event occurred (77%), with nearly 40% of them aged 80 and above. The average time to LA event from index date was 15 months. In terms of acid correction, 16 of the 30 (53%) received sodium bicarbonate, 17 (57%) received hemodialysis and 3 (10%) received both. Twenty‐four of the cases (80%) had a comorbidity that could have increased their risk of LA: liver dysfunction (14 persons), acute renal failure (7 persons), circulatory collapse (13 persons), severe infection (13 persons), dehydration (2 persons) and respiratory failure (12 persons). Seven patients received metformin within 30 days before the LA event date but most of these used metformin in combination with other diabetic medications. Fourteen of the 30 LA cases received only one class of diabetes medication. In the patients treated with metformin, the prescribed dose was between 500 mg and 1000 mg, and none of them had usage outside the suggested Japanese maximum dosage of 2250 mg.

The crude incidence rate of LA in all treated T2DM patients was 5.95 (95% CI: 4.16–8.51) per 100 000 patient‐years. The age‐sex adjusted incidence rates for metformin users and non‐users were 5.80 (95% CI: 2.68–12.57) per 100 000 and 5.78 (95% CI: 3.73–8.96) per 100 000 person‐years, respectively. Age‐sex adjusted incidence rates of LA in metformin users ranged from 4.46 (95% CI, 1.06–18.82) in patients with CLD to 23.59 (95% CI, 8.68–64.12) per 100 000 person‐years in patients with CKD. The incidence rate ratio (comparing metformin users to nonusers) was 1.00 (95% CI: 0.41–2.47), and within subgroups of patients with CLD or CKD was 0.86 (95% CI: 0.16–4.69) and 0.98 (95% CI: 0.30–3.23), respectively (Table 2).

**Table 2:** Incidences of LA— stratified by age, CLD and CKD

Exposure	Person‐years	N. of events	Crude incidence [95% CI]	Age‐sex adjusted incidence [95% CI]*	Adjusted rate ratio [95% CI] *
Target population	504 169	30	5.95 [4.16–8.51]		
Metformin users	133 614	7	5.24 [2.50–10.99]	5.80 [2.68–12.57]	1.00 [0.41–2.47]
Non metformin users	370 555	23	6.21 [4.12–9.34]	5.78 [3.73–8.96]	Ref
Age <65 y	192 796	7	3.63 [1.73–7.62]	3.60 [2.50–5.30]	
Metformin users	70 218	2	2.85 [0.71–11.39]	2.75 [0.62–12.13]	0.70 [0.14–3.57]
Non Metformin users	122 578	5	4.08 [1.70–9.80]	3.91 [1.59–9.62]	Ref
Age 65–74 y	166 470	10	6.01 [3.23–11.16]	5.90 [4.30–8.20]	
Metformin users	42 162	1	2.37 [0.33–16.84]	2.30 [0.29–17.91]	0.33 [0.04–2.54]
Non Metformin users	124 309	9	7.24 [3.77–13.91]	6.88 [3.53–13.44]	Ref
Age 75 y–	144 902	13	8.97 [5.21–15.45]	8.80 [6.60–11.80]	
Metformin users	21 234	4	18.84 [7.07–50.19]	18.75 [7.11–49.45]	2.58 [0.80–8.34]
Non Metformin users	123 668	9	7.28 [3.79–13.99]	7.27 [3.76–14.07]	Ref
Disease subgroup					
Patients without CLD					
Metformin users	103 550	5	4.83 [2.01–11.60]	5.25 [2.16–12.75]	1.09 [0.39–3.15]
Non Metformin users	285 830	16	5.60 [3.43–9.14]	4.80 [2.68–8.59]	Ref
Patients with CLD					
Metformin users	30 064	2	6.65 [1.66–26.60]	4.46 [1.06–18.82]	0.86 [0.16–4.69]
Non Metformin users	84 725	7	8.26 [3.94–17.33]	5.18 [1.58–16.97]	Ref
Patients without CKD					
Metformin users	115 212	3	2.60 [0.84–8.07]	2.81 [ 0.84–9.37]	1.52 [0.37–6.19]
Non Metformin users	305 164	6	1.97 [0.88–4.38]	1.85 [ 0.83–4.11]	Ref
Patients with CKD					
Metformin users	18 402	4	21.74 [8.16–57.92]	23.59 [ 8.68–64.12]	0.98 [0.30–3.23]
Non Metformin users	65 391	17	26.00 [16.16–41.82]	23.96 [14.01–40.98]	Ref

Data was shown as 100 000 person‐years.

* Poisson regression was adjusted for age groups (18–64, 65–74, 75–) and gender (female, male).

In the Cox proportional hazard models, the adjusted hazard ratio (HR) of metformin use was 0.92 (95% CI: 0.33–2.55) while the adjusted HR for having CKD was 7.33 (95% CI: 3.17–16.96) and for heart failure was 2.37 (95% CI: 0.98–5.75). In propensity score‐matched cohort, the adjusted HR for metformin use was 0.90 (95% CI: 0.26–3.11; Table 3).The sensitivity analysis results were similar to the main analyses. Among the new user cohort, the HR for metformin use was not appreciably changed compared with the full cohort. In addition, the hazard for metformin use showed the consistency across the sub‐cohorts after adjusted by stratum of propensity score (Table 4, Supplement Table 2–5).

**Table 3:** Hazard ratio of lactic acidosis for metformin by CLD/CKD

Variables	Main cohort (before matching)*	Main cohort adjusted by propensity score (20 strata)†	Propensity score‐matched cohort‡
**Number of LA cases**	30	30	10
Non metformin users	23	23	5
Metformin users	7	7	5
**Hazard ratio**			
Non metformin users	Ref	Ref	Ref
Metformin users	0.92 [0.33–2.55]	0.89 [0.32–2.49]	0.90 [0.26–3.11]
Age ≥ 75 y	1.67 [0.79–3.53]	1.36 [0.61–3.04]	2.02 [0.41–9.09]
Female	1.03 [0.49–2.17]	1.10 [0.52–2.30]	1.05 [0.29–3.77]
Duration of metformin use before cohort entry (day)	1.00 [1.00–1.01]	1.00 [1.00–1.01]	1.01 [1.00–1.01]
Insulin	1.64 [0.76–3.51]	—	2.85 [0.31–26.39]
Hypertension	1.26 [0.54–2.89]	—	1.03 [0.21–5.10]
Ischemic heart disease	1.11 [0.42–2.89]	—	0.96 [0.83–1.10]
Heart failure	2.37 [0.98–5.75]	2.39 [1.00–5.71]	—
Complications of diabetes	1.73 [0.78–3.84]	1.94 [0.90–4.19]	2.58 [0.67–10.01]
Dyslipidemia	—	—	0.48 [0.06–3.81]
Cerebrovascular disease	—	—	1.65 [0.20–13.73]
Gastric ulcer	2.05 [0.86–4.93]	—	1.64 [0.20–13.79]
Chronic liver disease	1.44 [0.65–3.17]	1.42 [0.65–3.13]	3.35 [0.97–11.58]
Liver Cirrhosis	—	—	—
Chronic kidney disease	7.33 [3.17–16.96]	7.11 [3.08–16.43]	23.12 [4.90–109]
Renal failure	—	—	—

* The covariates which caused at least a 10% shift in the risk estimate for the univariate analysis were adjusted in the final model. The COX model for each analysis was constructed separately.

† We categorized the continuous propensity score into 20 strata of 5% each for the distribution of scores. The covariates which caused at least a 10% shift in the risk estimate after given by stratum of propensity score were adjusted in the final model.

‡ Variables in propensity score matched cohort were adjusted for stratum of propensity score only, which were estimated using the same variables.

**Table 4:** Association between Metformin use, CKD and incident LA

Sub‐cohorts	No of events	Hazard ratio [95% CI]*	Hazard ratio [95% CI]† propensity score adjusted
**Association between Metformin use and incident LA**			
Patients with CLD	9	0.79 [0.12–5.19]	0.70 [0.10–4.76]
Patient without CLD	21	1.38 [0.26–7.23]	0.97 [0.28–3.31]
Patient with CKD	21	0.66 [0.18–2.45]	1.04 [0.33–3.28]
Patient without CKD	9	1.45 [0.29–7.33]	1.38 [0.27–6.96]
Patients without CKD or CLD	7	1.18 [0.23–6.19]	1.44 [0.27–7.76]
**Association between CKD and incident LA**			
Patients with CLD	9	14.27 [2.91–69.97]	14.79 [3.00–72.90]
Patient without CLD	21	5.04 [1.86–13.63]	4.79 [1.77–12.94]

* The covariates which caused at least a 10% shift in the risk estimate for the univariate analysis were adjusted in the final model. The COX model for each analysis was constructed separately.

† We categorized the continuous propensity score into 20 strata of 5% each for the distribution of scores. The covariates which caused at least a 10% shift in the risk estimate after given by stratum of propensity score were adjusted in the final model.

## Discussion

We found no association between use of metformin and risk of LA, but did find having CKD was a strong risk factor for LA. In addition, within patients with CKD, we found no increased risk of LA with metformin use. To our knowledge, this is the first epidemiology study to quantify and assess the risk of LA among treated Japanese diabetes patients. Clinical trials have typically excluded or been under‐representative of patients with old age, hepatic impairment and renal dysfunction, which have made it a challenge for the practitioners to choose the optimal diabetes treatment for these populations. This study, with 283 491 patients, is one of the largest studies to date, with the elderly representing over one‐third of the study population. It provides important information on the tolerability to metformin among these patient groups. The presence of CKD was associated with a seven‐fold increase in risk of LA, and is by far the strongest risk factor identified. These data show that metformin use does not play a role in LA. This is supported by a recent systematic review, which suggested no causal link between the use of metformin and LA.^14^

Of the few previous clinical trials that included those over 70 years of age, metformin treatment was not associated with significantly increased plasma lactate levels, as compared with other forms of treatment.^15^, ^16^ As LA is a multifactorial morbidity, it was expected that the advanced age would have been associated with increased risk. In our study, the overall incidence of LA increases with aging, but no significant difference was found between metformin users and non‐users after stratifying by age. Our finding better reflects real‐life metformin use than seen in clinical trials. As LA is a rare event, most trials were not powered to provide a precise estimate of incidence of LA, and particular among patients aged 75 or more. As metformin is the leading oral hypoglycemic agents and associated with a reduced odds of diabetes‐related complications, metformin is particularly encouraged in the older patients.^17^, ^18^ However, in the older diabetic patients a high proportion have impaired renal function, so it may be necessary to use dose reduction because of changes in several pharmacokinetic and pharmacodynamic parameters.^19^

Given the importance of the liver for lactate clearance, focusing on the severity and prognosis for the liver disease has been suggested. In general, previous case reports found that the underlying liver disease was described as alcoholic‐related, but cautioned that ‘metformin should be avoided in patients with clinical or laboratory evidence of hepatic disease’.^4^, ^20^ Risk aversion probably discouraged physicians from using metformin in patients with any liver disease. In our study, the incidence of LA and adjusted rate ratio show no relationship between risk of LA and use of metformin among patients with CLD. However, we cannot rule out the possibility that the indeterminate cause and degree of liver damage could mask an association because the metabolism pathway suggested that liver function may modify the risk of LA.^21^

Because metformin is eliminated by the kidneys, it may accumulate when renal function decreases, with the potential for exposure‐dependent toxicity that could precipitate lactate accumulation. In this regard, FDA listed renal impairment, defining by serum creatinine level, as precaution in the box warning, but the American Diabetes Association recommend that estimated GFR is a better measure of renal function when considering drug choice for renal safety.^22^ For the clinical guideline outside of the U.S., the threshold of estimated GFR varied from 60 ml/min/1.73 m^2^ up to stage 5 CKD, albeit widely, the most frequently recommended for withdrawing metformin were at 30 ml/min/1.73 m^2^.^23^ Likewise, in addition to avoiding using metformin among patients with critical illness, the Japanese Diabetes Society/Japan Association for Diabetes Education and Care suggested that metformin should be used with caution among certain populations such as the elderly, and patients with CKD, CLD, reduced pulmonary function, dehydration and uncontrolled alcohol intake etc.^24^

A recent study based on a large‐scale registry found that the use of metformin may significantly reduce the mortality among metformin users less than 80 years, and those with moderate renal failure (estimated GFR between 30 and 60 ml/min). The benefits of metformin use might outweigh its risk in certain populations.^17^ One previous study showed many physicians prescribed metformin to patients with stage 3 and 4 CKD and rarely found the metformin‐induced LA.^25^ In our study, among the patients with CKD the rate of LA in patients taking metformin was the same as the rate of LA in patients who did not take metformin, implying that metformin use was concomitant but not causative. In terms of the development of LA, the presence of comorbid conditions is most likely responsible for the LA.^8^ Our study supported the observation that a significant increase in risk for LA is associated with CKD, rather than with metformin. Unfortunately, this study was unable to evaluate how the CKD stage affects the risk of LA because of lack of estimated GFR data. Current clinical guidance allows metformin to be used in patients with estimated GFR as low as 30 ml/min/1.73 m^2^, with lower doses if the estimated GFR is lower than 45 ml/min/1.73 m^2^. 22 As for the careful monitoring, it is no doubt that metformin should be withdrawn in those with poor tolerance.

This study was limited by its observational design and confounding by unmeasured factors cannot be rule out. However, the consistence between the models suggested the robustness of primary finding. As diabetes might have started before database membership, we were not able to assess duration of diabetes for the patients with prevalent diabetes, nor capture the care when they visited facilities which did not contact with MDV company. The lack of increased risk in our new user sub‐cohort and in the several sub‐cohorts suggests that duration was not an unmeasured confounding factor. In addition, we treated the use of metformin as time‐varying exposure throughout study period to minimize the immortal time bias and misclassification bias. The risk of exposure misclassification may have been present if the patients received the treatment or dispensed their drugs in the healthcare institutions not covered by the study database. Moreover, left truncation of data is unlikely to bias the association between metformin and LA in an immediate exposure effect.^26^ As the medical conditions of the patients included in this database might be more severe than those treated in primary care only, it is possible that we overestimate the true background incidence of LA. The particular strength of this study was the use of propensity score matching to ensure balance in baseline characteristic between metformin users and non‐metformin users, reducing the risk of treatment allocation bias. It was not possible to validate the diagnoses of LA because data privacy requirements prevent the linking of these data to source documents. However, the morbidities were identified based on medical claims data, and most were accompanied by acute events such as acute renal failure, circulatory collapse and sepsis, which are independent risk factors of LA.^27^ The clinical course suggested that the cases experienced the correction of acidemia were captured appropriately. Furthermore, the consistent results in the new user cohort suggested that survival bias is limited in the current study. Last, caution should be exercised to extrapolate our results to patients who did not receive any drug treatment for glycemic control, and those with worsening of renal function.

In conclusion, our finding shows low incidence of LA among adult patients with type 2 diabetes mellitus treated with anti‐diabetes drug user, but that risk increased with older age and presence of CKD in both metformin and non‐metformin anti‐diabetes drug users. The use of metformin was not associated with risk of LA in patients with T2DM including those with CKD or CLD. It seemed reasonable to conclude that the CKD is an important determinant of LA, and that metformin use is likely not a risk factor.

## Conflict of Interest

All authors are employees of Takeda Pharmaceutical Company limited.

This study was presented at the 31^st^ International Conference of Pharmacoepidemiology and Therapeutic Risk Management August 22–26, 2015 in Boston, MA, USA.

**Key Points**

- The crude incidence of lactic acidosis in the Japanese Type 2 diabetes population treated with anti‐diabetes drugs was low and increased with increasing age and in the presence of chronic kidney disease (CKD).
- The use of metformin was not associated with increased risk of lactic acidosis, even in patients with CKD or chronic liver disease.
- The presence of CKD may independently increase risk of lactic acidosis by seven‐fold, regardless of metformin use.

## Ethical Approval of Studies

The study protocol was reviewed and approved by the Japan Epidemiological Association.

## Supporting information

Appendix: Epidemiology of Lactic Acidosis in Type 2 Diabetes Patients with Metformin in Japan

Supporting info item
