# Supplementary material for: Epidemiology of lactic acidosis in type 2 diabetes patients with metformin in Japan
Source: Pharmacoepidemiol Drug Saf. 2016 May 25;25(10):1196–203. doi: 10.1002/pds.4030 (PMC5089598; doi:10.1002/pds.4030)
Supplement: Supplementary file 1 — Supporting info item [file PDS-25-1196-s001.docx]

**Appendix: Epidemiology of Lactic Acidosis in Type 2 Diabetes Patients with Metformin in Japan**

[Suppl. Table 1 Incidences of LA (new user cohort) 2](#_Toc447721691)

[Suppl. Table 2 Unadjusted and Adjusted Risk of Lactic acidosis for Metformin by CLD/CKD (new user cohort) 3](#_Toc447721692)

[Suppl. Table 3 Hazard ratio of Lactic acidosis among patient with and without CLD given adjusted by propensity score (20 strata) 4](#_Toc447721693)

[Suppl. Table 4 Hazard ratio of Lactic acidosis among patient with and without CKD given adjusted by propensity score (20 strata) 6](#_Toc447721694)

[Suppl. Table 5 Hazard ratio of Lactic acidosis among patient without CLD or CKD given adjusted by propensity score (20 strata) 8](#_Toc447721695)

Suppl. Table 1 Incidences of LA (new user cohort)

| Exposure | Person-years | N. of events | Crude incidence  [95% CI] | Age-sex adjusted incidence [95% CI]* | Adjusted rate ratio [95% CI] * |
| --- | --- | --- | --- | --- | --- |
| Target population | 282,062 | 15 | 5.32 [3.21- 8.82] |  |  |
| Metformin users | 73,834 | 3 | 4.06 [1.31-12.60] | 4.39 [1.37-14.08] | 0.84 [0.20-3.54] |
| Non Metformin users | 208,228 | 12 | 5.76 [3.27-10.15] | 5.21 [2.64-10.29] | Ref |
| Age <65 y | 99,815 | 3 | 3.01 [0.97-9.32] | 2.90 [0.97- 8.70] |  |
| Metformin users | 36,524 | 0 |  |  | -- |
| Non Metformin users | 63,291 | 3 |  |  | -- |
| Age 65-74 y | 95,585 | 5 | 5.23 [2.18-12.57] | 5.00 [2.10-11.90] |  |
| Metformin users | 24,239 | 0 |  |  | -- |
| Non Metformin users | 71,346 | 5 |  |  | -- |
| Age 75 y- | 86,662 | 7 | 8.08 [3.85-16.94] | 7.60 [2.50-22.70] |  |
| Metformin users | 13,071 | 3 | 22.95 [7.40- 71.17] | 22.80 [7.13-72.88] | 4.24 [0.96-18.72] |
| Non Metformin users | 73,591 | 4 | 5.44 [2.04 -14.48] | 5.38 [2.07-14.00] | Ref |
| Disease subgroup |  |  |  |  |  |
| Patient without CLD |  |  |  |  |  |
| Metformin users | 55,226 | 2 | 3.62 [0.91 -14.48] | 3.61 [0.94-13.94] | 1.15 [0.18-7.19] |
| Non Metformin users | 153,821 | 6 | 3.90 [1.75-8.68] | 3.15 [1.01- 9.87] | Ref |
| Patient with CLD |  |  |  |  |  |
| Metformin users | 18,609 | 1 | 5.37 [0.76-38.15] | 4.36 [0.59-32.01] | 0.58 [0.05-6.31] |
| Non Metformin users | 54,407 | 6 | 11.03 [4.95-24.55] | 7.50 [2.03-27.78] | Ref |
| Patient without CKD |  |  |  |  |  |
| Metformin users | 64,999 | 1 | 1.54 [0.22-10.92] | 1.68 [0.23-12.10] | 1.02 [0.07-14.41] |
| Non Metformin users | 169,100 | 3 | 1.77 [0.57-5.50] | 1.64 [0.41- 6.57] | Ref |
| Patient with CKD |  |  |  |  |  |
| Metformin users | 8,835 | 2 | 22.64 [ 5.66-90.51] | 23.59 [5.69-97.77] | 1.15 [0.21-6.42] |
| Non Metformin users | 39,128 | 9 | 23.00 [11.97-44.20] | 20.54 [9.12-46.24] | Ref |

Note, Data was shown as 100,000 person-years

* Poisson regression was adjusted for age groups (18-64, 65-74, 75-), and gender (female, male).

Suppl. Table 2 Unadjusted and Adjusted Risk of Lactic acidosis for Metformin by CLD/CKD (new user cohort)

| Model | Variable | Overall | Patient with CLD | Patient with CKD |
| --- | --- | --- | --- | --- |
| Unadjusted HR [95% CI] |  |  |  |  |
|  | Non metformin users | ref | ref | ref |
|  | Metformin users | 0.68 [0.19-2.40] | 0.46 [0.06-3.81] | 0.94 [0.20-4.36] |
| Adjusted HR [95% CI]* |  |  |  |  |
|  | Non metformin users | ref | ref | ref |
|  | Metformin users | 1.48 [0.39-5.58] | 0.98 [0.11-8.83] | 1.13 [0.24-5.43] |
|  | Age>=75 y | 1.61 [0.57-4.60] | 1.76 [0.38-8.05] | 1.68 [0.50-5.58] |
|  | Female | 1.13 [0.40-3.21] | 0.30 [0.04-2.48] | 0.97 [0.28-3.34] |
|  | Hypertension | 1.23 [0.37-4.04] | -- | - |
|  | Ischemic heart disease | 2.51 [0.71-8.79] | -- | 6.26 [1.82-21.55] |
|  | Heart failure | 1.44 [0.40-5.22] | -- | -- |
|  | Complication of Diabetes | 1.21 [0.38-3.85] | -- | -- |
|  | Gastric ulcer | 5.09 [1.69-15.30] | 11.48 [2.52-52.39] | 3.12 [0.79-12.24] |
|  | Malignancy | -- | -- | -- |
|  | Metastatic Cancer | -- | -- | -- |
|  | Chronic liver disease | 2.01 [0.65-6.20] | -- | 2.26 [0.69-7.45] |
|  | Liver Cirrhosis | 2.88 [0.55-15.21] | 2.50 [0.47-13.38] | -- |
|  | Chronic kidney disease | 11.56 [3.48-38.36] | 11.07 [2.13-57.55] | -- |

* The covariates which caused at least a 10% shift in the risk estimate for the univariate analysis were adjusted in the final model. The COX model for each analysis was constructed separately.

Suppl. Table 3 Hazard ratio of Lactic acidosis among patient with and without CLD given adjusted by propensity score (20 strata)

| Variables | Patients with CLD*  (Main cohort) | Patient with CLD†  (Adjusted by PS) | Patients without CLD*  (Main cohort) | Patient without CLD†  (Adjusted by PS) |
| --- | --- | --- | --- | --- |
| **Number of LA cases** | 9 | 9 | 21 | 21 |
| Non metformin users | 7 | 7 | 16 | 16 |
| Metformin users | 2 | 2 | 5 | 5 |
| **Hazard ratio** |  |  |  |  |
| Non metformin users | ref | ref | ref | ref |
| Metformin users | 0.79 [0.12- 5.19] | 0.70 [0.10- 4.76] | 1.38 [0.26-7.23] | 0.97 [0.28-3.31] |
| Age>=75 y | 1.39 [0.34-5.74] | 1.67 [0.35- 7.98] | 1.77 [0.73-4.33] | 1.42 [0.54-3.75] |
| Female | 0.23 [0.03-1.18] | 0.22 [0.03- 1.74] | 1.69 [0.71-4.04] | 1.76 [0.73-4.20] |
| Duration of metformin use  before cohort entry (day) | 1.00 [1.00-1.01] | 1.00 [1.00- 1.01] | 1.00 [1.00-1.01] | 1.00 [1.00-1.01] |
| Insulin | -- | -- | -- | -- |
| Hypertension | -- | -- | 1.17 [0.44-3.12] | -- |
| Ischemic heart disease | -- | -- | -- | -- |
| Heart failure | -- | -- | 3.40 [1.25-9.28] | 3.06 [1.13-8.27] |
| Complications of diabetes | -- | -- | 3.36 [1.28-8.84] | 3.46 [1.36-8.81] |
| Dyslipidemia | -- | -- | -- | -- |
| Cerebrovascular disease | -- | -- | -- | -- |
| Biliary disease |  | -- | -- | -- |
| Obesity |  | -- | -- | -- |
| Gastric ulcer | 6.44 [1.69-24.53] | 7.61 [1.76- 32.89] | -- | -- |
| Malignancy | -- | -- | -- | -- |
| Metastatic Cancer | -- | -- | -- | -- |
| Chronic liver disease | -- | -- | -- | -- |
| Liver Cirrhosis | 1.97 [0.40- 9.82] | 2.52 [0.40- 15.91] | -- | -- |
| Chronic kidney disease | 14.27 [2.91-69.97] | 14.79 [3.00- 72.90] | 5.04 [1.86-13.63] | 4.79 [1.77-12.94] |
| Renal failure | -- | -- | -- | -- |

* The covariates which caused at least a 10% shift in the risk estimate for the univariate analysis were adjusted in the final model. The COX model for each analysis was constructed separately.

† We categorized the continuous propensity score into 20 groups of 5% each for the distribution of scores. The covariates which caused at least a 10% shift in the risk estimate after given by stratum of propensity score were adjusted in the final model.

Suppl. Table 4 Hazard ratio of Lactic acidosis among patient with and without CKD given adjusted by propensity score (20 strata)

| Variables | Patients with CKD*  (Main cohort) | Patient with CKD†  (Adjusted by PS) | Patients without CKD*  (Main cohort) | Patient without CKD†  (Adjusted by PS) |
| --- | --- | --- | --- | --- |
| **Number of LA cases** | 21 | 21 | 9 | 9 |
| Non metformin users | 17 | 17 | 6 | 6 |
| Metformin users | 4 | 4 | 3 | 3 |
| **Hazard ratio** |  |  |  |  |
| Non metformin users | ref | ref | ref | ref |
| Metformin users | 0.66 [0.18-2.45] | 1.04 [0.33- 3.28] | 1.45 [0.29-7.33] | 1.38 [0.27-6.96] |
| Age>=75 y | 1.91 [0.79-4.61] | 1.58 [0.61- 4.10] | 1.07 [0.25-4.49] | 0.62 [0.14-2.80] |
| Female | 0.96 [0.40-2.34] | 1.01 [0.42- 2.47] | 1.29 [0.34-4.93] | 1.51 [0.40-5.75] |
| Duration of metformin use  before cohort entry (day) | 1.00 [1.00-1.01] | -- | 1.01 [1.00-1.01] | 1.01 [1.00-1.01] |
| Insulin | -- | -- | 3.45 [0.81-14.7] | -- |
| Hypertension | - | -- | 2.27 [0.53-9.67] | -- |
| Ischemic heart disease | 2.79 [1.02-7.65] | 2.81 [1.04- 7.57] | -- | -- |
| Heart failure | 1.95 [0.67-5.63] | -- | 3.18 [0.70-14.51] | -- |
| Complications of diabetes | -- | -- | -- | -- |
| Dyslipidemia | -- | -- | -- | -- |
| Cerebrovascular disease | -- | -- | -- | -- |
| Biliary disease | -- | -- | -- | -- |
| Obesity | -- | -- | -- | -- |
| Gastric ulcer | -- | -- | 2.73 [0.63-11.82] | -- |
| Malignancy | -- | -- | -- | -- |
| Metastatic Cancer | -- | -- | -- | -- |
| Chronic liver disease | 1.56 [0.63-3.90] | 1.56 [0.63-3.88] | 1.00 [0.21- 4.90] | 0.90 [0.19- 4.38] |
| Liver Cirrhosis | -- | -- | -- | -- |
| Chronic kidney disease | -- | -- | -- | -- |
| Renal failure | 0.29 [0.07-1.27] | 0.23 [0.05-1.06] | -- | -- |

* The covariates which caused at least a 10% shift in the risk estimate for the univariate analysis were adjusted in the final model. The COX model for each analysis was constructed separately.

† We categorized the continuous propensity score into 20 strata of 5% each for the distribution of scores. The covariates which caused at least a 10% shift in the risk estimate after given by stratum of propensity score were adjusted in the final model.

Suppl. Table 5 Hazard ratio of Lactic acidosis among patient without CLD or CKD given adjusted by propensity score (20 strata)

| Model | Patients without CLD or CKD* | Patient without CLD or CKD†  (Adjusted by PS) |
| --- | --- | --- |
| Number of LA cases | 7 | 7 |
| Non metformin users | 4 | 4 |
| Metformin users | 3 | 3 |
| Adjusted HR [95% CI]* |  |  |
| Non metformin users | ref | ref |
| Metformin users | 1.18 [0.23- 6.19] | 1.44 [0.27- 7.76] |
| Age>=75 y | 1.11 [0.20- 6.05] | 0.70 [0.11- 4.40] |
| Female | 1.95 [0.43- 8.86] | 2.24 [0.48- 10.346] |
| Duration of metformin use  before cohort entry (day) | 1.01 [1.00- 1.01] | 1.01 [1.00- 1.01] |
| Insulin | -- | -- |
| Hypertension | -- | -- |
| Ischemic heart disease | -- | -- |
| Heart failure | -- | -- |
| Complications of diabetes | 4.62 [0.99-21.6] | 4.31 [0.92-20.17] |
| Dyslipidemia | 4.38 [0.95-20.4] | 4.09 [0.88-18.96] |
| Cerebrovascular disease | -- | -- |
| Biliary disease | -- | -- |
| Obesity | -- | -- |
| Gastric ulcer | -- | -- |
| Malignancy | -- | -- |
| Metastatic Cancer | -- | -- |
| Chronic liver disease | -- | -- |
| Liver Cirrhosis | -- | -- |
| Chronic kidney disease | -- | -- |
| Renal failure | -- | -- |

* The covariates which caused at least a 10% shift in the risk estimate for the univariate analysis were adjusted in the final model. The COX model for each analysis was constructed separately.

† We categorized the continuous propensity score into 20 strata of 5% each for the distribution of scores. The covariates which caused at least a 10% shift in the risk estimate after given by stratum of propensity score were adjusted in the final model.
